# EMDLP: Ensemble multiscale deep learning model for RNA methylation site prediction

**DOI:** 10.1186/s12859-022-04756-1

**Published:** 2022-06-08

**Authors:** Honglei Wang, Hui Liu, Tao Huang, Gangshen Li, Lin Zhang, Yanjing Sun

**Affiliations:** 1 Engineering Research Center of Intelligent Control for Underground Space, Ministry of Education, China University of Mining and Technology, Xuzhou, 221116 China; 2 School of Information and Control Engineering, China University of Mining and Technology, Xuzhou, 221116 China; 3 School of Information Engineering, Xuzhou College of Industrial Technology, Xuzhou, 221400 China

**Keywords:** RNA modification site, Deep learning, Natural language processing, Predictor

## Abstract

**Background:**

Recent research recommends that epi-transcriptome regulation through post-transcriptional RNA modifications is essential for all sorts of RNA. Exact identification of RNA modification is vital for understanding their purposes and regulatory mechanisms. However, traditional experimental methods of identifying RNA modification sites are relatively complicated, time-consuming, and laborious.

Machine learning approaches have been applied in the procedures of RNA sequence features extraction and classification in a computational way, which may supplement experimental approaches more efficiently. Recently, convolutional neural network (CNN) and long short-term memory (LSTM) have been demonstrated achievements in modification site prediction on account of their powerful functions in representation learning. However, CNN can learn the local response from the spatial data but cannot learn sequential correlations. And LSTM is specialized for sequential modeling and can access both the contextual representation but lacks spatial data extraction compared with CNN. There is strong motivation to construct a prediction framework using natural language processing (NLP), deep learning (DL) for these reasons.

**Results:**

This study presents an ensemble multiscale deep learning predictor (EMDLP) to identify RNA methylation sites in an NLP and DL way. It organically combines the dilated convolution and Bidirectional LSTM (BiLSTM), which helps to take better advantage of the local and global information for site prediction.

The first step of EMDLP is to represent the RNA sequences in an NLP way. Thus, three encodings, e.g., RNA word embedding, One-hot encoding, and RGloVe, which is an improved learning method of word vector representation based on GloVe, are adopted to decipher sites from the viewpoints of the local and global information. Then, a dilated convolutional Bidirectional LSTM network (DCB) model is constructed with the dilated convolutional neural network (DCNN) followed by BiLSTM to extract potential contributing features for methylation site prediction. Finally, these three encoding methods are integrated by a soft vote to obtain better predictive performance. Experiment results on m^1^A and m^6^A reveal that the area under the receiver operating characteristic(AUROC) of EMDLP obtains respectively 95.56%, 85.24%, and outperforms the state-of-the-art models. To maximize user convenience, a user-friendly webserver for EMDLP was publicly available at http://www.labiip.net/EMDLP/index.php (http://47.104.130.81/EMDLP/index.php).

**Conclusions:**

We developed a predictor for m^1^A and m^6^A methylation sites.

**Supplementary Information:**

The online version contains supplementary material available at 10.1186/s12859-022-04756-1.

## Background

**Fig. 1:**
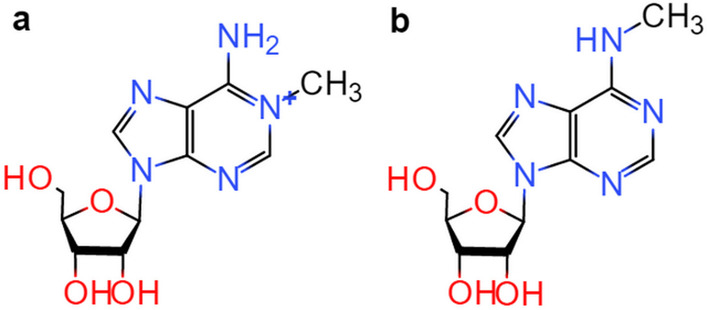
Chemical structures of modifications. **a** m^1^A modification. **b** m^6^A modification

RNA molecules’ functional diversity is enriched by post-transcriptional RNA modifications, which regulate all stages of RNA life [1]. Up to now, there are around 160 different forms of RNA modifications that have been discovered [2], including N^1^-methyladenosine(m^1^A), N^6^-methyladenosine(m^6^A), 5-methylcytosine(m^5^C), N^2^-methylguanosine(m^2^G), 7-methylguanosine(m^7^G) [3, 4], etc. Among them, m^1^A modification is a prevalent RNA modification, which occurs on the nitrogen-1 position of the adenine base attached with a methyl group [5], as shown in Fig. 1a. It’s linked to problems with the respiratory chain, neurodevelopmental regression, and mediate antibiotic resistance bacteria, etc. [6–8]. Another modification affecting adenine is m^6^A modification, the most abundant modification in mammals, which occurs on the nitrogen-6 position of the adenosine base [9], as shown in Fig. 1b. It has a profound impact on human growth and disease [10]. The adenosine usually undergoes m^1^A and m^6^A [11]. Interestingly, m^1^A is also known to undergo Dimroth rearrangement to m^6^A under alkaline conditions [11]. Therefore, it is important to accurately identify m^1^A and m^6^A modification sites to uncover the mechanisms and functions of those modifications [12].

Many experimental methods for identifying m^1^A and m^6^A modification sites have been constructed with the significant advances in high-throughput sequencing technology, such as m^6^A-CLIP [13], m^6^A-miCLIP [14], m^1^A-seq [15], m^1^A-ID-seq [11], etc. However, the experimental methods are expensive and time-consuming, which limit their extensive use [16]. Fortunately, various computational methods have become powerful supplements in this area.

Most machine learning methods designed for site prediction from sequences usually first extracted features based on human-understood feature methods, followed by a classifier to predict whether the site is a methylation site or not. For example, RAMPred extracted features based on nucleotide chemical properties (NCP), nucleotide composition (NC), and adopted the support vector machine (SVM) to predict the m^1^A methylation site for the first time [17]. iRNA-3typeA extracted features based on NCP, accumulated nucleotide frequency(ANF), and adopted SVM to predict m^1^A, m^6^A, and A-to-I modification sites [18]. iMRM extracted features based on NCP, NC, One-hot encoding, Dinucleotide Binary Encoding (DBE), Nucleotide Density (ND), Dinucleotide physicochemical properties (DPCP) and adopted eXtreme Gradient Boosting(XGboost) to predict m^1^A, m^6^A, m^5^C, $\psi$ and A-to-I modification sites, whose performance was superior to existing methods [19]. M^6^AMRFS extracted features based on DBE, ANF, used the F-score algorithm combined with Sequential Forward Search(SFS) to raise feature representation, and employed XGBoost to predict m^6^A site [20]. RNAMethPre extracted the features of the flanking sequences, the local secondary structure data, and the relative position data first, then adopted SVM to predict m^6^A methylation site with satisfactory performance [21]. SRAMP combines three random forest classifiers by exploiting One-hot encoding, K-nearest neighbor encoding, and Nucleotide pair spectrum encoding to predict m^6^A sites [22]. RFAthM^6^A extracted features based on four encoding methods, including Knucleotide frequencies (KNF), position-specific nucleotide sequence profile (PSNSP), Kspaced nucleotide pair frequencies (KSNPF), and position-specific dinucleotide sequence profile (PSDSP), respectively, then built four random forest models, which were competitive compared with AthMethPre, M^6^ATH, and RAM-NPPS [23]. WHISTLE adds 35 genomic features in addition to integrating conventional sequence features and predicts m^6^A methylation by SVM [24], which significantly improved compared to other computational approaches. However, genomic features are not always available when only a few RNA sequences are provided to predict m^6^A methylation. These conclusions show that extracted features is extremely critical to the final prediction.

It is well known that RNA-seq contains rich biometric information. Thus, the Rational representation of RNA sequences becomes even more critical. To address this problem, representation learning of sequences by natural language processing (NLP) has attracted a lot of attention [25], where an RNA sequence is regarded as a sentence, and a *k*-monomeric unit (*k*-mer) is regarded as a word, has gained great traction [26, 27]. Compared with conventional machine learning methods, most of the deep learning(DL) models can be divided into three parts: first, learning input data representations by NLP models [28]; second, composing over the word vectors that have been learned [29]; third, classing by a classifier to predict whether or not the site is a methylation site.

By far, some prediction methods using NLP and DL networks have been developed to predict m^6^A or m^1^A sites. Among them, Gene2Vec [30], DeepPromise [12], and EDLm^6^Apred [16] were the most representative and advanced methods for methylation site prediction. Specifically, Gene2Vec was developed to predict m^6^A site based on Word2vec [31] and convolutional neural network (CNN). DeepPromise adopted CNN and integrated enhanced nucleic acid content (ENAC) [32], RNA word embedding [33], and One-hot encoding [20, 34] features to identify m^1^A and m^6^A sites. EDLm^6^Apred adopted Word2vec, One-hot encoding, RNA word embedding, and BiLSTM to predict m^6^A sites. However, the existing methods have the following shortcomings. As is known, from the perspective of NLP, ENAC, One-hot, and RNA word embedding focused on the local semantic information [16] but ignored the context and global information. Word2vec encoding considered the context window information, ignoring the global information [35]. From the perspective of DL, CNN can learn the local response from the spatial data [25]. The different scale of the convolution kernel impacts the network's learning ability. Gene2Vec [30] and DeepPromise [12] directly used CNN composed of a single-scale convolution kernel, which might lead to incomplete representation learning of sequences [36]. The missing information in both methods may be important to the final site prediction. In addition, CNN has no memory function and lacks the ability to learn sequential correlations [25]. On the contrary, EDLm^6^Apred [16] presented a deep BiLSTM network to address the above issue, which simultaneously accessed context information. However, BiLSTM lacks spatial data extraction compared with CNN and needs a high training time [37, 38].

Consider the above questions. This paper proposes EMDLP to identify RNA methylation sites in an NLP and DL way. Specifically, One-hot encoding, RNA word embedding, and RGloVe were initially used to encode the sequences. Secondly, the DCB model was constructed with DCNN followed by BiLSTM to extract potential contributing features for methylation site prediction. Third, Three predictors were constructed based on the DCB model by the three feature encoding methods above. Finally, EMDLP was formulated by a soft vote with average predicted probabilities to use the three predictors to obtain better predictive performance. The results showed that the performance of the EMDLP model outperformed the state-of-the-art methods such as DeepPromise [12] and EDLm^6^Apred [16] in independent tests.

## Results

### Evaluation metrics

To estimate the prediction of the models, we adopted widely used binary classifier evaluation metrics, including Sensitivity(Sn, Recall), Specificity(Sp), Accuracy(Acc), Precision(Pre), F1 score (F1), Matthews correlation coefficient(MCC), Area under the receiver operating characteristic(AUROC), and Area under the precision-recall curve (AUPRC). Sn, Sp, Acc, Pre, F1, MCC are defined as follows:

1 $$Sn = \frac{TP}{{TP + FN}}$$

2 $$Sp = \frac{TN}{{TN + FP}}$$

3 $$Acc = \frac{TP + TN}{{TP + TN + FP + FN}}$$

4 $$Pre = \frac{TP}{{TP + FP}}$$

5 $$F1 = 2 \times \frac{Precision \times Recall}{{Precision + Recall}}$$

6 $$MCC = \frac{TP \times TN - FP \times FN}{{\sqrt {(TP + FP) \times (TP + FN) \times (TN + FP) \times (TN + FN)} }}$$

where TP refers to true positive, TN refers to true negative, FP refers to false positive, and FN refers to false negative. In addition, the AUROC and AUPRC values are calculated based on the receiver operation curve (ROC) and the precision-recall curve (PRC), respectively. All the metric values range from 0 to 1 except for the MCC value, which ranges lies in [− 1, + 1], with a higher value indicating better performance.

### Results analysis

This paper first examined the performance of RGloVe and GloVe on different sliding window sizes. Second, the self-built DCB model was compared and analyzed with the CNN, DCNN, and BiLSTM models. Third, this study compared the RGloVe feature encoding with the three others on predicting methylation modification sites. Last, this paper compared the EMDLP model with state-of-the-art methods based on the independent datasets. Our computing device has two NVIDIA RTX2080Ti GPU and 11 GB of GPU device memory. In addition to the GPU, the machine has two 2.3 GHz 16-core Intel(R) Xeon(R) Gold 5218 CPU and 128 GB of RAM. The device is installed with 64-bit Windows10 Professional Edition 20H2, python 3.7.6, Keras 2.2.4, and TensorFlow-gpu 1.14.0.

The size of the sliding window is an important parameter that affects the performance of the encoding scheme. Based on benchmark datasets, this experiment compares the performance of RGloVe and GloVe in predicting m^1^A and m^6^A methylation sites under four different sliding window sizes(i.e., 8, 15, 30, and 60). RGloVe is based on the GloVe model framework and adopts RMSProp instead of Adagrad to minimize the loss function of the global vector model. As a result, RGloVe shows the best prediction performance when the sliding window length = 30, as shown in Table 1. The experiment results show that using RMSProp can train the model more effectively.

**Table 1:** AUROC scores of RGloVe and GloVe under different sliding windows sizes based on benchmark datasets

Modification type	Encoding	Window sizes = 8	Window sizes = 15	Window sizes = 30	Window sizes = 60
m^1^A	RGloVe	0.9283	0.9317	**0.9377**	0.9315
	GloVe	0.9282	0.9193	0.9305	0.9185
m^6^A	RGloVe	0.8414	0.8415	**0.8432**	0.8407
	GloVe	0.8399	0.8420	0.8414	0.8372

The bolded values represent the best results

### Comparison with other different learning models

Next, DCB was compared and analyzed with CNN, DCNN, and BiLSTM using the same benchmark datasets. The experiments used RGloVe encoding to describe the RNA sequence, constructed CNN_RGloVe_, DCNN_RGloVe_, BiLSTM_RGloVe_, and DCB_RGloVe_, respectively. Among them, CNN_RGloVe_ employed the CNN model in Deeppromise [12]. DCB_RGloVe_ represented a self-built DCB model, including the DCNN and BiLSTM stage. The DCNN_RGloVed_ denoted the DCB_RGloVe_ removing the BiLSTM stage, which was substituted by the flatten layer. Similarly, the BiLSTM_RGloVe_ represented the DCB_RGloVe_ without the DCNN stage.

**Fig. 2:**
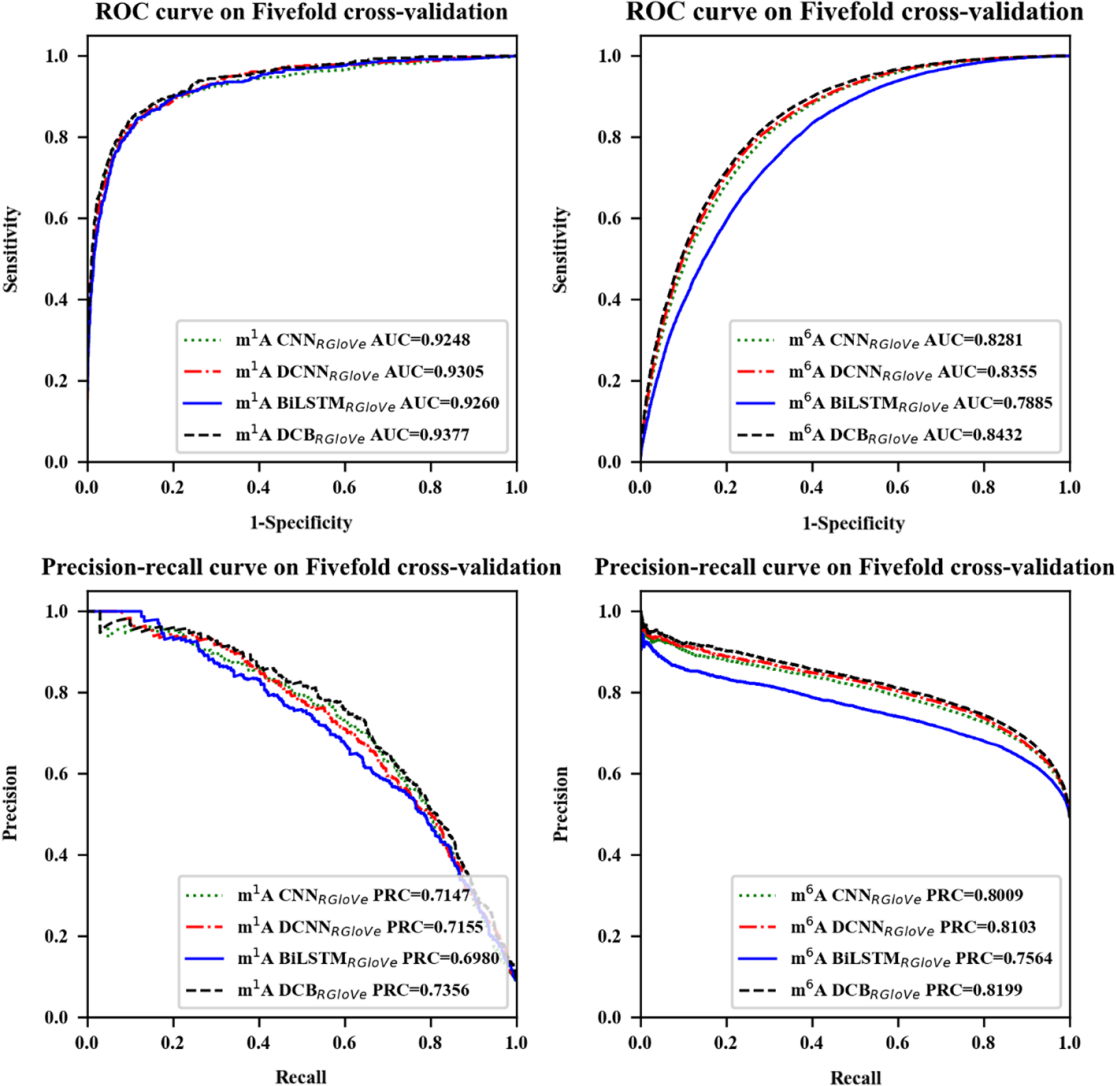
Performance of the different models through fivefold cross-validation. The models are CNN_RGloVe_, DCNN_RGloVe_, BiLSTM_RGloVe_, and DCB_RGloVe,_ respectively. "CNN_RGloVe_" employs the CNN model in Deeppromise; "DCB_RGloVe_" represents a self-built DCB model, including the DCNN and the BiLSTM stage; "DCNN_RGloVe_" denotes the DCB_RGloVe_ removing the BiLSTM stage; "BiLSTM_RGloVe_" represents the DCB_RGloVe_ without the DCNN stage

The fivefold cross-validation evaluation results, the AUROC and AUPRC curves on the m^1^A and m^6^A are shown in Fig. 2 and Table 2. The result shows the AUROC of DCNN_RGloVe_ is 0.57% and 0.74% higher than CNN_RGloVe_’s on m^1^A and m^6^A, and the AUPRC of DCNN_RGloVe_ is 0.08% and 0.94% higher than CNN_RGloVe_’s. This result.

**Table 2:** Evaluation results of the different models trained on the fivefold cross-validation

Modification type	Classifiers	AUROC	Acc (%)	Sn (%)	Sp (%)	MCC (%)	Pre (%)	F1 (%)	AUPRC	Time (s)
m^1^A	CNN_RGloVe_	0.9248	94.06	**66.95**	96.78	63.97	67.52	67.23	0.7147	127
	DCNN_RGloVe_	0.9305	94.22	58.01	97.84	61.97	72.88	64.60	0.7155	96
	BiLSTM_RGloVe_	0.9260	93.02	66.44	95.68	59.63	60.62	63.40	0.6980	2104
	DCB_RGloVe_	**0.9377**	**94.62**	61.72	**97.91**	**65.04**	**74.69**	**67.59**	**0.7356**	1809
m^6^A	CNN_RGloVe_	0.8281	74.93	81.84	68.22	50.47	71.44	76.29	0.8009	5264
	DCNN_RGloVe_	0.8355	75.79	82.48	69.29	52.18	72.29	77.05	0.8103	18,732
	BiLSTM_RGloVe_	0.7885	71.42	**83.87**	59.33	44.48	66.70	74.31	0.7564	131,340
	DCB_RGloVe_	**0.8432**	**76.46**	79.30	**73.65**	**53.03**	**74.96**	**77.07**	**0.8199**	21,638

The bolded values represent the best results

Verifies that the single-scale convolution kernel in CNN is challenging to learn deep semantics from RNA sequences. On the contrary, the multiscale convolution kernels can extract additional features to provide deep semantics.

In addition, the study compared the performance of DCB_RGloVe_ and DCNN_RGloVe_. The AUROC of DCB_RGloVe_ is 0.72% and 0.77% higher than DCNN_RGloVe_’s on m^1^A and m^6^A, respectively, and the AUPRC of DCB_RGloVe_ is 2.01% and 0.96% higher than DCNN_RGloVe_’s on m^1^A and m^6^A, respectively. The reason may be that DCNN has no memory function and cannot learn sequential correlations. On the contrary, DCB can capture the local correlation of different spatial structures according to DCNN and effectively learn the context of each *k*-mer in the text according to BiLSTM. In summary, DCB can understand sequence semantics more accurately than other methods.

Finally, the study compared the running time of DCB_RGloVe_ and BiLSTM_RGloVe_. Although many factors affect the model's training time, the experiment results show that the training time of BiLSTM_RGloVe_ is very long, for it is several times that of DCB_RGloVe_. The reason is that the max-pooling layer of the DCNN stage reduces the parameters of the network, which plays an active role in lowering dimensionality and computational complexity.

In conclusion, the DCB_RGloVe_ classifier could effectively and quickly capture the sequence details on m^1^A and m^6^A modification sites.

### Comparison with other different feature encoding methods

**Fig. 3:**
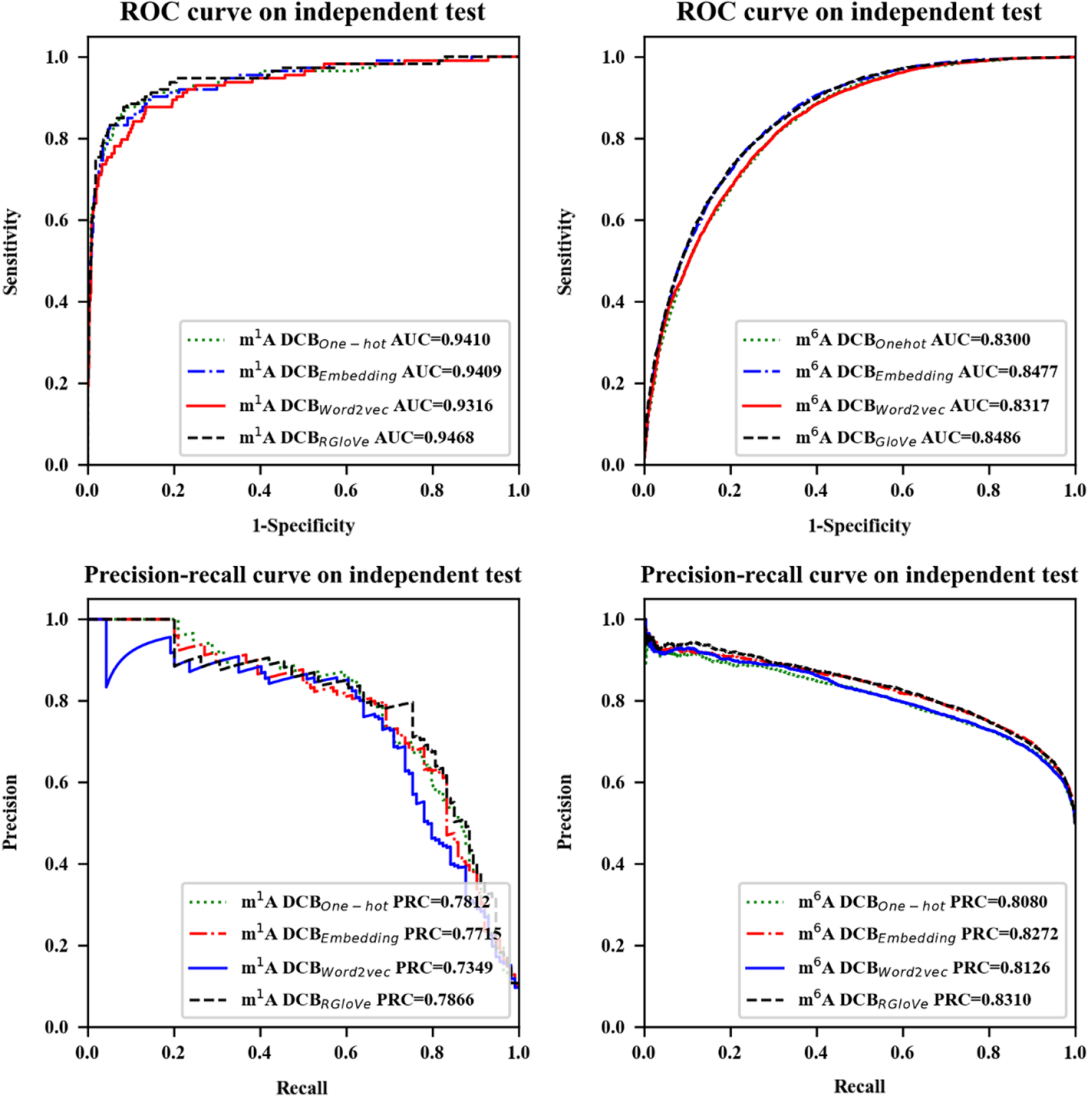
Performance of the DCB model based on One-hot encoding, RNA word embedding, Word2vec, and RGloVe

Besides, the following content compared the prediction performance of the four feature encoding methods. The experiment encoded the sequences by our RGloVe and the three commonly used schemes, RNA word embedding, One-hot encoding, and word2vec, respectively, then applied the same DCB model to predict the modification site on the same independent dataset. The comparison results demonstrate that RGloVe outperforms the other three encoding techniques in predicting AUROC, as shown in Fig. 3 and Table 3. In the sense of exactly, for m^1^A and m^6^A sites, DCB_RGloVe_ achieved AUROC 0.9468 and 0.8486 and more accurately than other methods. The reason is that the One-hot encoding and RNA word embedding emphasize local semantic information, and Word2vec encoding highlights the context windows information, but the above three encodings ignore the global information. RGloVe inherits the advantages of GloVe, which combines the benefits of global matrix factorization and local context approaches [37]. Therefore, RGloVe can improve the model prediction accuracy according to this advantage.

**Table 3:** Evaluation results of the DCB model based on One-hot encoding, RNA word embedding, Word2vec, and RGloVe

Modification type	Classifiers	AUROC	Acc (%)	Sn (%)	Sp (%)	MCC (%)	Pre (%)	F1 (%)	AUPRC
m^1^A	DCB_One-hot_	0.9410	95.37	64.04	98.51	69.66	81.11	71.57	0.7812
	DCB_Embedding_	0.9409	95.37	**65.79**	98.33	70.0	79.79	**72.12**	0.7715
	DCB_word2vec_	0.9316	95.29	61.4	**98.68**	68.72	**82.35**	70.35	0.7349
	DCB_RGloVe_	**0.9468**	**95.45**	64.04	98.6	**70.12**	82.02	71.92	**0.7866**
m^6^A	DCB_One-hot_	0.8300	74.51	72.25	**76.76**	49.06	**75.57**	73.87	0.8080
	DCB_Embedding_	0.8477	**76.52**	83.30	69.79	**53.56**	73.28	77.97	0.8272
	DCB_word2vec_	0.8317	75.10	79.60	70.62	50.43	72.95	76.13	0.8126
	DCB_RGloVe_	**0.8486**	76.36	**84.2**	68.57	53.41	72.72	**78.04**	**0.8310**

The bolded values represent the best results

In summary, RGloVe shows higher semantic accuracy than the other three commonly used schemes.

### Comparison with state-of-the-art approaches

Finally, EMDLP was compared with other state-of-the-art approaches on the same independent datasets, such as DeepPromise [12] and EDLm^6^Apred [16]. To make the comparison more illustrative, we built DCB_DeepPromise_ by replacing the CNN model in DeepPromise with DCB, and our EMDLP replaced the ENAC encoding in DCB_DeepPromise_ with RGloVe.

**Fig. 4:**
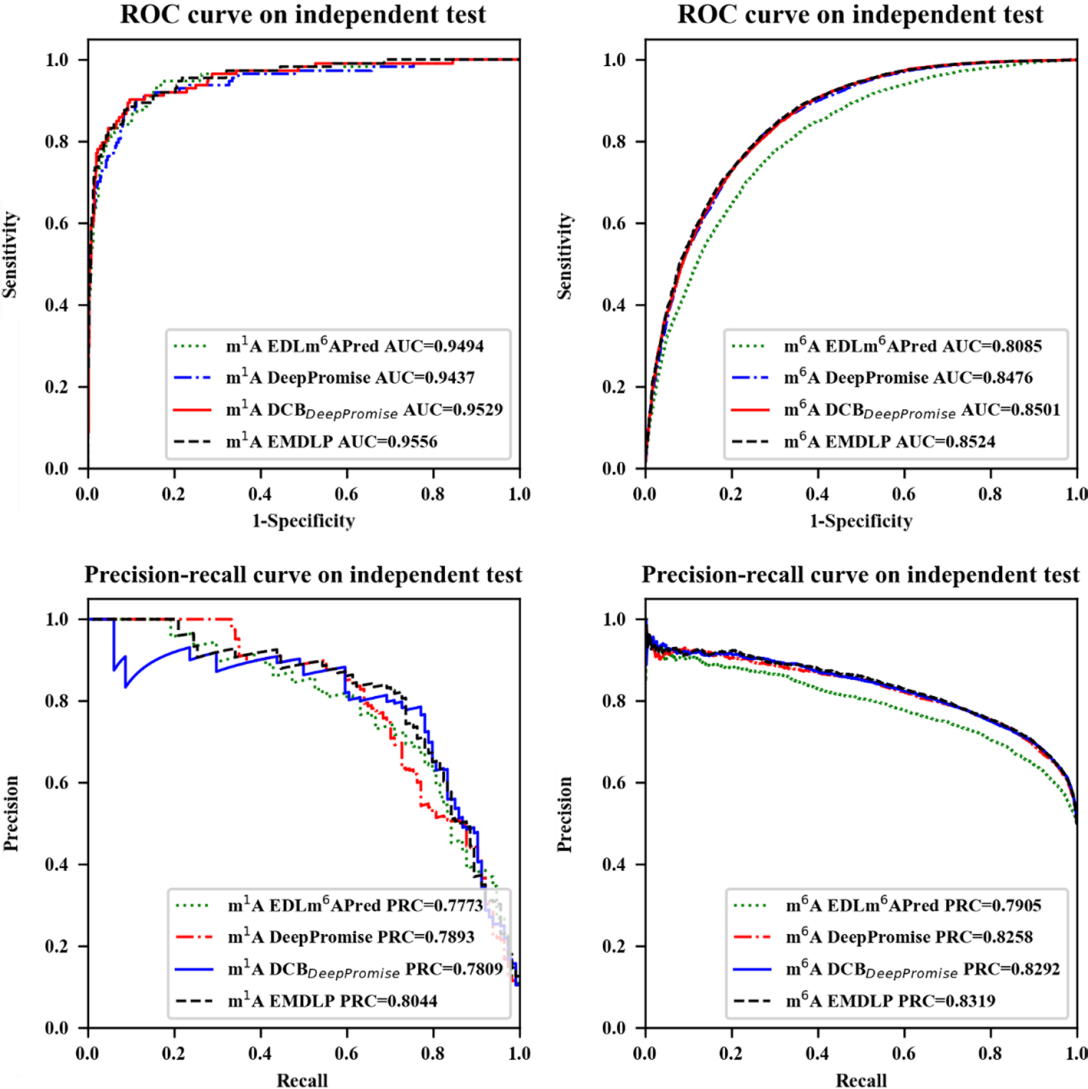
Performance of EMDLP and other methods on the independent test

**Fig. 5:**
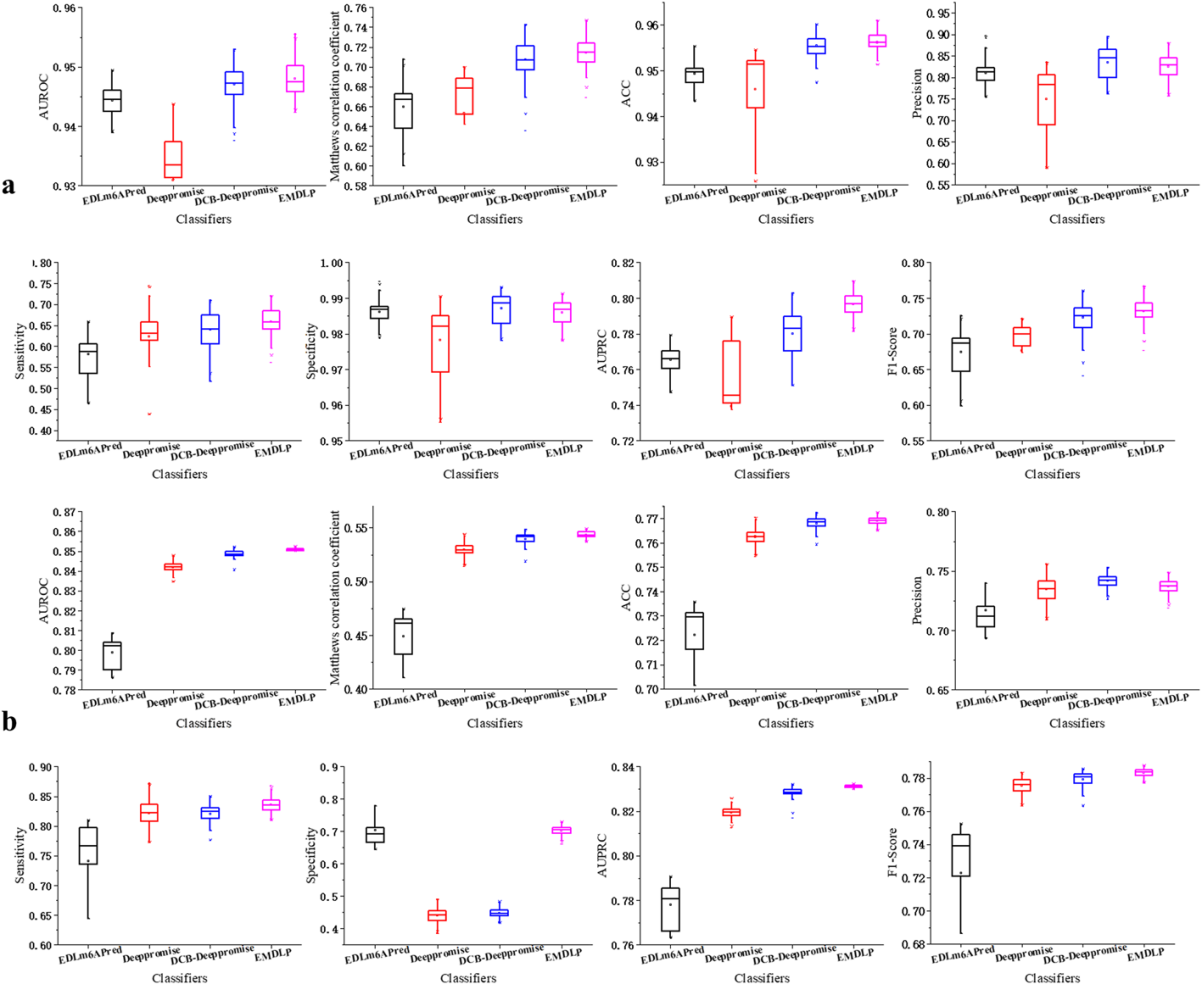
Boxplot of eight metrics for comparative performance assessment of the four methods based on the pAerformance of 100 replications of four methods. **a** for the m^1^A independent dataset. **b** for the m^6^A independent dataset

In order to evaluate the reliability of the model, the EDLm^6^Apred, DeepPromise, DCBDeepPromise, and EMDLP models were performed 100 replicate experiments on the same independent test sets of m^1^A and m^6^A, respectively. In each replicate, new evaluation results were produced. As shown in Fig. 4, Table 4, and Fig. 5, the AUROC and AUPRC of EMDLP are better than other approaches. The reason may be that ENAC, One-hot, and RNA word embeddings focus on local semantic information, and Word2vec encoding considers context window information, but none of them pay attention to global statistical information. At the same time, RGloVe can represent semantic information sequences more comprehensively than the other four encodings. And DCB is more suitable for extracting the RNA sequence's features than the other methods. Furthermore, We test the statistical significance of AUROC values between different tools by the student’s *t*-test [39], as shown in Table 5.

**Table 4:** Compare EMDLP model

Modification type	Classifiers	AUROC	Acc (%)	Sn (%)	Sp (%)	MCC (%)	Pre (%)	F1 (%)	AUPRC
m^1^A	EDLm^6^Apred	0.9494	95.06	64.91	98.07	68.10	77.08	70.47	0.7773
	DeepPromise	0.9437	95.30	65.79	98.25	69.57	78.95	71.77	0.7893
	DCB_DeepPromise_	0.9529	95.61	**67.54**	98.42	**71.67**	81.05	**73.68**	0.7809
	EMDLP	**0.9556**	**95.62**	61.40	**99.04**	70.69	**86.42**	71.79	**0.8044**
m^6^A	EDLm^6^APred	0.8085	73.38	80.14	66.66	47.23	70.52	75.02	0.7905
	DeepPromise	0.8476	**77.07**	82.15	45.00	54.43	**74.79**	78.30	0.8258
	DCB_DeepPromise_	0.8501	76.76	81.89	44.95	53.81	74.19	77.85	0.8292
	EMDLP	**0.8524**	76.98	**84.36**	**69.64**	**54.58**	73.44	**78.52**	**0.8319**

The bolded values represent the best results

**Table 5:** Statistically significant correlation matrix for the difference in the performance of the four classifiers

Modification type	Classifiers	Classifiers			
		EDLm^6^APred	DeepPromise	DCB_DeepPromise_	EMDLP
m^1^A	EDLm^6^APred				
	DeepPromise	6.80137E-27			
	DCB_DeepPromise_	2.14723E-11	5.22548E-34		
	EMDLP	8.734E-20	4.51535E-37	0.01606677	
m^6^A	EDLm^6^APred				
	DeepPromise	1.7731E-122			
	DCB_DeepPromise_	3.3248E-133	2.05181E-42		
	EMDLP	8.6672E-142	6.72773E-87	3.06352E-20	

### Webserver

**Fig. 6:**
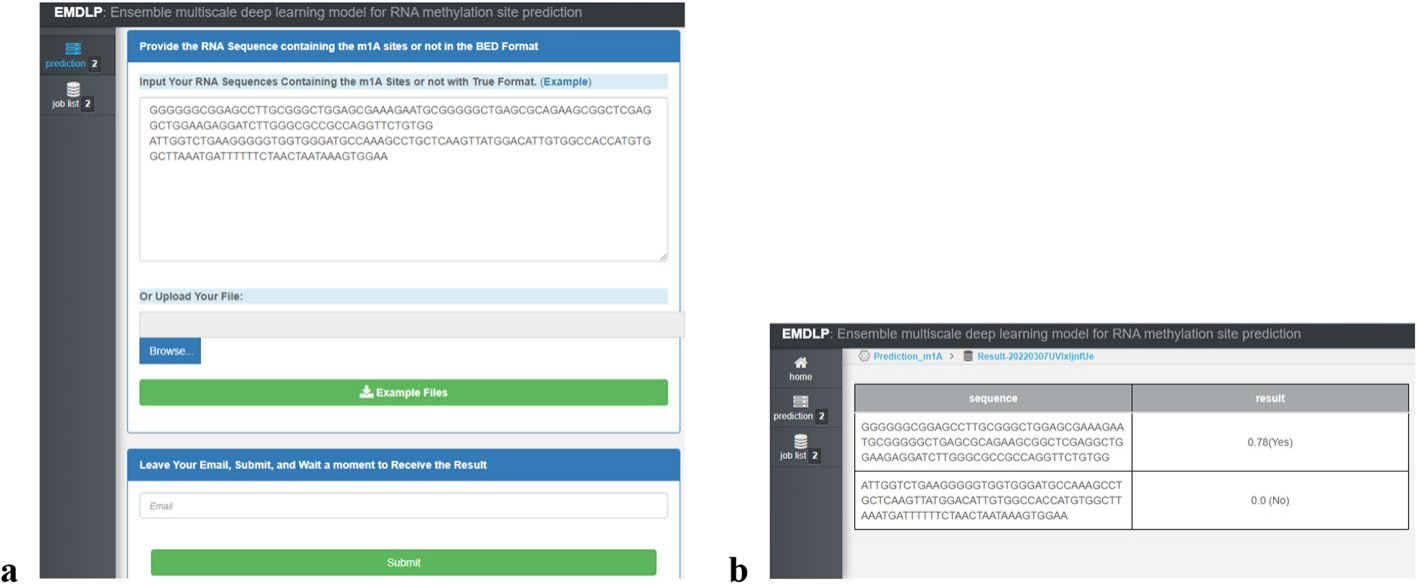
Screenshot of EMDLP webserver. **a** Site input interface of EMDLP. **b** The prediction result returned by EMDLP

We established an online webserver to simultaneously identify m^1^A and m.^6^A modifications in H. sapiens to facilitate scientific research. The user-friendly webserver for EMDLP was publicly available at http://www.labiip.net/EMDLP/index.php (http://47.104.130.81/EMDLP/index.php). The usage guide of the webserver for EMDLP is as follows. Open the home page at http://www.labiip.net/EMDLP/index.php (http://47.104.130.81/EMDLP/index.php). First, clicking the "Prediction" button and selecting the "m^1^A" or"m^6^A" successively, the page will appear, as shown in Fig. 6a. Second, Type or paste an RNA sequence in the input box. Third, leave your email in the input box, clicking the "submit" button, and the predictive results will appear on a new page, as shown in Fig. 6b.

## Discussion

This paper proposes EMDLP to identify RNA methylation sites in an NLP and DL way. The specific discussion is as follows:

Firstly, this study compared the performance of predicting m^1^A and m^6^A methylation sites under four different sliding window sizes (i.e., 8, 15, 30, and 60) based on the RGloVe and GloVe encoding methods. The evaluation results show that using RMSProp instead of Adagrad to minimize the loss function of the global vector model can indeed train the model more effectively. This result is consistent with that of Ruder, S. (2017), who pointed out that RMSProp can overcome the weakness of Adagrad. RGloVe shows the best prediction performance when the sliding window length = 30.

Secondly, based on the feature representation of the sequence by the above RGloVe, this study compared the DCB model with the CNN, DCNN, and BiLSTM models for predicting methylation modification sites. The experiment result shows the AUROC of DCNN_RGloVe_ is 0.57% and 0.74% higher than CNN_RGloVe_'s on m^1^A and m^6^A. This study confirms that the multiscale convolution kernels can extract different features to provide deep semantics. The experiment results show that the training time of BiLSTM_RGloVe_ is very long, and it is several times that of DCB_RGloVe_. That also accords with Min, X.’s conclusion, which showed that the max-pooling layer of the DCNN stage reduces the parameters of the network, which plays an active role in lowering dimensionality and computational complexity. The experimental results show that the DCB_RGloVe_ model is superior to other models in predicting m^1^A and m^6^A sites. This study confirms that the combination of DCNN and BiLSTM makes the understanding of sequence semantics more accurate.

Third, based on the above self-built DCB model, this paper compared the prediction performance of RGloVe, RNA word embedding, One-hot encoding, and word2vec. The results reveal that Our RGloVe outperforms the other three encoding schemes in prediction performance. This finding is consistent with Pennington, J (2014), who proposed that GloVe shows higher semantic accuracy than word2vec.

Finally, EMDLP was constructed by a soft vote to use the three predictors to obtain better predictive performance. This paper compared the prediction performance of EMDLP, DeepPromise, DCB_DeepPromise_, and EDLm^6^Apred based on the independent datasets. The results show that the AUROC of EMDLP is significantly better than the three methods. This study further indicates that RGloVe can better represent the semantic information of sequences than the other four encodings, and DCB is more suitable for extracting the RNA sequence's features than the other methods.

## Conclusions

The contribution of this paper proposes a predictor EMDLP to identify RNA methylation sites by NLP and DL way. It organically combines the dilated convolution and BiLSTM, which helps take better advantage of the local and global information for site prediction.

Although EMDLP outperforms state-of-the-art predictors, which is currently limited to humans and has not been extended to other model organisms due to the lack of a sufficient number of single-nucleotide datasets for other species. It is worth looking forward to testing the performance of EMDLP when sufficient other species RNA modification datasets become available in the future.

## Materials and methods

### Datasets

We have extracted two common types of human RNA modification site datasets published at single-nucleotide resolution, including m^1^A and m^6^A. For the m^1^A and m^6^A sites, the datasets in this paper were derived from the previous studies of Chen et al. [12] and Zou et al. [30], respectively. The only difference is that the Zou validation set was used as the independent test set of this paper on the m^6^A site.

The study divided the dataset into two parts: a benchmark dataset for cross-validation testing and an independent dataset for independent testing. It took the modified/non-modified site as the center for each sample and brought the (2n + 1)-nt partial sequence window. It was worth noting that the "n" for these two modifications was different. Referring to the experimental results in Chen’s paper, the size of the optimal window was 101 and 1001 for m^1^A and m^6^A sites[12], respectively. If the length of the original sequences were shorter than 2n + 1, the empty positions would be filled with the character "-" to ensure the sequence length is consistent. The ratio of positive and negative samples of m^1^A sites and m^6^A sites was 1:10 and 1:1, respectively. The statistic of these two RNA modification datasets is shown in Table 6.

**Table 6:** A statistical of these two RNA modification datasets

Modification type	Dataset	Window size	Number of positive samples	Number of negative samples
m^1^A	m^1^A_BM	101	593	5930
m^1^A	m^1^A_IND	101	114	1140
m^6^A	m^6^A_ BM	1001	26,586	27,371
m^6^A	m^6^A_IND	1001	6879	6914

*BM* benchmark; *IND* independent

### Feature encoding representation on different perspectives

As we all know, feature encoding is the key to evaluating the excellent performance of site prediction models. This paper encodes the sequences by RNA word embedding, One-hot encoding, and RGloVe.

One-hot encoding is a sparse binary, high-dimensional word vector, while RNA word embedding is a continuous, low-dimensional dense word vector that captures the local semantic information. RGloVe inherits the principle of GloVe, which captures the global semantic information.

One-hot encoding is a very simple encoding method to describe the nucleotides sequence. The four nucleotides and the the gap symbol "-" are encoded as $\sum { = \{ {\text{A}},{\text{C}},{\text{G}},{\text{T}}, - \} }$, where A = (1,0,0,0,0), C = (0,1,0,0,0), G = (0,0,1,0,0), T = (0,0,0,1,0), and "-" = (0,0,0,0,1). Take m^1^A as an example, a sequence of 101nts is transformed to 505-bit vectors.

RNA word embedding is a standard method for encoding RNA sequences. A sliding window of size k slides on the RNA sequence by overlapping an equal length to form a *k*-mer sub-sequence, and these sub-sequences are created as a vocabulary. Take m^1^A as an example. A sequence of 101nts is converted to 99 sub-sequence through a sliding window of size 3. The study obtained 105 different sub-sequences, which are indexed by a unique integer index. Each pre-processed sequence is changed with an integer index and fed into the Keras embedding layer to generate 300-dimension word vectors. Thus, the 101nts sequences are transformed into a matrix of 99 × 300.

RNA word embedding only considers the frequency information but neglects the context and global information. Word2vec only trains independently by information from each local context window, while it does not use the statistical data in the global co-occurrence matrix [35]. Pennington et al. [40] proposed global vectors(GloVe) that can consider the statistical data in the global co-occurrence matrix and used Adagrad to train GloVe word embeddings [41]. But, Adagrad has a primary weakness, which can cause the learning rate of Adagrad to decrease and get extremely small, at which point the algorithm can not learn new information [41]. Therefore, the study uses RMSProp instead of Adagrad to minimize the loss function of the global vector model. The word vector trained by this method is called RGloVe. The specific analysis process is as follows.

The statistics of *k*-mer incidence is the most important data source for learning embedding representations. *Y* denotes the matrix of co-occurrence counts, and *Y*_*ij*_ records the frequency of the word *k*-mer $j$ appearing in the context sliding windows of the word *k*-mer *i*. $i,\,j \in \left[ {1,\,W} \right]$ are two *k*-mer indexes, the vocabulary size *W* = 105. According to the GloVe model, we get the embedding vector by training the cost function under,

7 $$K = \sum\limits_{i,\,j = 1}^{W} {f(Y_{ij} )({\mathbf{e}}_{i}^{T} \widetilde{{\mathbf{e}}}_{j} + b_{i} + \widetilde{b}_{j} - \log Y_{ij} )^{2} }$$

where $e \in \mathbb{R}^{D}$ are expected embedding vectors, ${\mathbf{\tilde{e}}} \in \mathbb{R}^{D}$ are separate context *k*-mer vectors that help obtain ${\mathbf{e}}$, $b,\,\widetilde{b} \in {\mathbb{R}}$ are the biases for ${\mathbf{e}},\,\widetilde{{\mathbf{e}}}$ respectively. $f(y)$ is a non-decreasing weighting function below

8 $$f(y) = \left\{ {\begin{array}{*{20}c} {(y/y_{\max } )^{\beta } \begin{array}{*{20}c} {} & {if\begin{array}{*{20}c} {} & {y^{{}} { < }^{{}} y_{\max } } \\ \end{array} } \\ \end{array} } \\ {1\begin{array}{*{20}c} {} & {} & {} & {{\text{otherwise}}} \\ \end{array} } \\ \end{array} } \right.$$

where $y_{\max }$ is a maximum cutoff value and $\beta$ denotes the fractional power scaling, which is commonly 0.75.

The original GloVe uses Adagrad [42] to minimize Eq. (7). At every time step $t$, the specific iterative rules are as follows:

9 $$z_{{t,{\kern 1pt} i}} = \nabla_{{\phi_{t} }} F(\phi_{t,\,i} )$$

where $z_{t,\,i}$ indicates the gradient of the objective function, $\phi_{t,\,i}$ is the parameter at a time step $t$. The Adagrad update for every parameter $\phi_{t,\,i}$ at each time step $t$ are as follows:

10 $$\phi_{t + 1,\,i} = \phi_{t,\,i} - \frac{\alpha }{{\sqrt {Z_{t,\,ii} + \delta } }} \cdot z_{t,\,i}$$

where $\alpha$ indicates the learning rate, $Z_{t,\,ii} \in {\mathbb{R}}^{d \times d}$ is a diagonal matrix where each diagonal element *i*, *i* is the sum of the gradients' squares. $\phi_{t,\,i}$ up to time step *t*, δ is commonly 1 $e$ − 8.

The primary deficiency of Adagrad is its accumulation of the squared gradients in the denominator, at which point the algorithm stops learning new information [41]. The RMSprop algorithm solves this flaw by reducing its monotonically decreasing learning rate. RMSprop does not accumulate all past square gradients but limits the window of accumulated past gradients to a fixed size $\xi$. The total of gradients is recursively defined as a decaying average of all past square gradients rather than merely keeping $\xi$ previous square gradients [41]. At time step $t$, the running average $E\left[ {z^{2} } \right]_{t}$ depends on the previous average $E\left[ {z^{2} } \right]_{{t{ - 1}}}$ and the current gradient $z_{t}^{2}$:

11 $$E\left[ {z^{2} } \right]_{t} = \lambda E\left[ {z^{2} } \right]_{t - 1} + (1 - \lambda )z_{t}^{2}$$

at each time step $t$, the RMSprop update for every parameter $\phi_{t}$ below:

12 $$\phi_{t + 1} = \phi_{t} - \frac{\alpha }{{\sqrt {E\left[ {z^{2} } \right]_{t} + \delta } }} \cdot z_{t}$$

The momentum term $\lambda$ is usually set to 0.9 or a similar value, while the learning rate of RMSprop $\alpha$ is 0.001. We use RMSprop to minimize Eq. (7) and obtained the D-dimensional embedding vector representations ${\mathbf{e}}_{1} ,{\mathbf{e}}_{2} ,{\mathbf{e}}_{3} , \ldots {\mathbf{e}}_{W} \in \mathbb{R}^{D}$. According to the vectors, the study has completed the embedding encoding of representation learning $f_{embedding} (x):{\mathbb{C}}^{L} \mapsto {\mathbb{R}}^{L \times D}$ by embedding each *k*-mer into the vector space ${\mathbb{R}}^{D}$:

13 $$f_{embedding} ({\mathbf{x}}) = [{\mathbf{e}}_{{x_{1} }} ,{\mathbf{e}}_{{x_{2} }} ,{\mathbf{e}}_{{x_{3} }} , \ldots {\mathbf{e}}_{{x_{L} }} ]$$

where ${\mathbf{x}} = [x_{1} ,x_{2} ,x_{3} , \ldots ,x_{L} ] \in \mathbb{C}^{L}$. We carried out the convolution stage based on the output $L \times D$ matrix.

Take m^1^A as an example. If the dimension is 300, the 101nts sequences are transformed into a matrix of 99 × 300. Three feature encoding input and output formats are in Table 7.

**Table 7:** Input and output formats with three kinds of feature encoding

Modification type	Encoding method	Input	Output
m^1^A	One-hot	101 × 1	101 × 5
	RNA word embedding	99 × 3	99 × 300
	RGloVe	99 × 3	99 × 300
m^6^A	One-hot	1001 × 1	1001 × 5
	RNA word embedding	999 × 3	999 × 300
	RGloVe	999 × 3	999 × 300

### Dilated convolutional neural network

Holschneider et al. [43] were the first to develop dilated convolution, which kept the feature map's resolution by introducing holes into the regular convolution [44]. Compared to ordinary convolution, dilated convolution adds a hyperparameter named dilation rate(DR), which corresponds to the number of kernel intervals, such as DR = 1 in ordinary convolution.

When applied to a one-dimensional situation, dilated convolution can be calculated as Eq. (14). Different dilution rates can be regarded as inserting varying sizes of blank rows between each kernel of convolution, as shown in Additional file 1: Fig. S1.

14 $$y_{j}^{{}} = f(\sum\limits_{n = 1}^{N} {x_{j + r*n} \omega_{n} } + b)$$

where *x*_*j*_ is the *j*th element of input, *y*_*j*_ denotes the output of the *j*th element in the DCNN, $\omega$ is the weight of the filter, *N* is the length of the filter, *r* is known as the DR.

In addition to the dilated convolution, the DCNN comprises the pooling and dropout layer. The pooling layer is applied to each feature map and outputs the average or maximum value of the input in a pooling window so that the pooling layer can reduce the number of parameters.

The dropout layer is used to avoid overfitting during model training and is the most commonly used regularization technique. In each training activity during forwarding propagation, some neurons are randomly set to zero, which intuitively leads to the integration of different networks. The dropout rate is the probability of a neuron withdrawing.

In this study, dilated convolutional layers of three dilation rates(DR = 1, 2, and 3, respectively) are concatenated to send to the BiLSTM stage.

### Bidirectional LSTM

BiLSTM is a specific sort of recurrent neural network(RNN) that combines forward LSTM and backward LSTM. Among them, forward LSTM calculates the hidden features in the forward direction and saves the output at each moment $\overrightarrow {{h_{2} ,}} \,\overrightarrow {{h_{{3}} }} ,\,...\overrightarrow {{h_{5} }}$. With the same reasoning, backward LSTM calculates the hidden features in the reverse direction and saves the output at each moment $\overleftarrow {{h_{5} }} ,\,\overleftarrow {{h_{4} }} ,\,...\overleftarrow {{h_{2} }}$, as shown in Additional file 1: Fig. S2. Ultimately, the final result is derived from merging the output values of the forward and backward LSTM layers at each instant.

The LSTM [45] framework addresses the exploding or disappearing gradients in RNNs. Commonly, the LSTM unit is defined as a current input $x_{t}$, a memory unit $C_{t}$, an input modulation vector $\widetilde{{C_{t} }}$, a hidden state $h_{t}$, a forget gate $f_{t}$, an input gate $i_{t}$, and an output gate $o_{t}$ at the moment $t$, as shown in Additional file 1: Fig. S3.

Among them, a memory unit $C_{t}$ is controlled by three "gates": a forget gate $f_{t}$, an input gate $i_{t}$, and an output gate $o_{t}$, where their entries are in [0, 1]. The following are the LSTM transition equations:

15 $$f_{t} = \sigma (W^{f} x_{t} + U^{f} h_{t - 1} + b^{f} )$$

16 $$i_{t} = \sigma (W^{i} x_{t} + U^{i} h_{t - 1} + b^{i} )$$

17 $$\tilde{C}_{t} = \tanh (W^{c} x_{t} + U^{c} h_{t - 1} + b^{c} )$$

18 $$C_{t} = f_{t} * C_{t - 1} + i_{t} * \tilde{C}_{t}$$

19 $$o_{t} = \sigma (W^{o} x_{t} + U^{o} h_{t - 1} + b^{o} )$$

20 $$h_{t} = o_{t} * \tanh (C_{t} )$$

where $W$ and $U$ are the weight metrics, $b$ represents bias, $\sigma$ is the logistic Sigmoid function, $*$ represents element-wise multiplication.

LSTM has been demonstrated significant benefits in modeling time series data attributable to features of its engineer. BiLSTM combines forward and backward LSTM, which overcomes the vanishing or exploding gradients and evaluates the context's meaning [25].

### Site prediction based on dilated convolutional Bidirectional LSTM

**Fig. 7:**
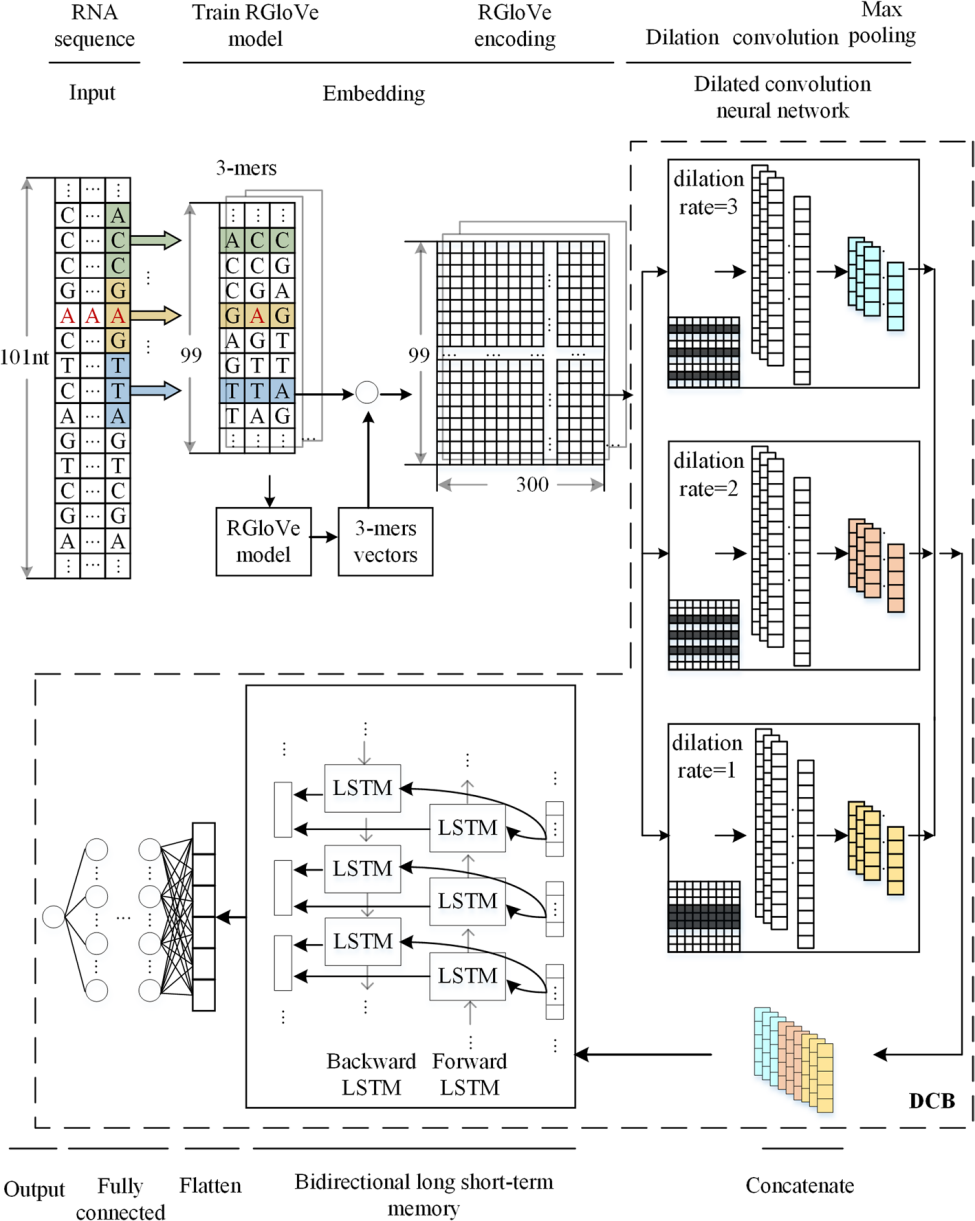
structure of our computational framework based on RGloVe, DCNN, and BiLSTM neural network to predict m^1^A methylation site

The study combined the DCB model with three encoding methods: RNA word embedding, one-hot encoding, and RGloVe to create three modification site predictors. Consider the RGloVe predictor, as shown in Fig. 7.

Suppose that we have N RNA sequences of *L*_*0*_-length. Each has a binary label indicating whether it is a methylation modification site, meaning *N*-labeled samples $\{ {\mathbf{x}}_{n} ,y_{n} \}_{n = 1}^{N}$ $y_{n} \in \left\{ {0,\,1} \right\}$. For each sequence ${\mathbf{x}}_{n}$ with A, C, T, G nucleotides, and "-", we split it into sub-sequences by using a split window. Each sub-sequence containing *k* nucleotides is called the *k*-mer motif. We extract the sub-sequence of length *k* with stride *s*, resulting in a *k*-mer motif of length $L = [(L_{0} - k)/s] + 1$. Take m^1^A as an example. A sequence of *L*_*0*_ = 101nts is converted to 99 sub-sequence through a split window of size *k* = 3 and stride *s* = 1, where all these 3-mers have a positive integer index in the set ${\mathbb{C}}$ = [1, 2, 3, 4…, 105], and sequence data ${\mathbf{x}} \in \mathbb{C}^{L}$.

The following content will specifically introduce learning a feature map $f:{\mathbb{C}}^{L} \mapsto {\mathbb{R}}^{d}$ that maps ${\mathbf{x}} \in \mathbb{C}^{L}$ into feature vectors ${\mathbf{h}} \in \mathbb{R}^{d}$ useful for DL tasks.

We used DCB with *k*-mer embedding to train the model, as shown in Fig. 7. The representation learning function $f:{\mathbb{C}}^{L} \mapsto {\mathbb{R}}^{d}$ can be separated into four stages:

21 $${\mathbf{h}} = f\left( x \right) = f_{BiLSTM} \left( {f_{concat} \left( {f_{DCNN} \left( {f_{embedding} \left( {\mathbf{x}} \right)} \right)} \right)} \right)$$

The embedding stage calculates the co-occurrence statistics of *k*-mers and maps them to the D-dimensional space ${\mathbb{R}}^{D}$.

The DCNN stage has three blocks of DCNNs, and the dilution rate of three DCNNs is 1, 2, and 3, respectively. A dilated convolutional layer with the rectified linear unit (ReLU) as its active function, a max-pooling layer, and a dropout unit are all included in each DCNN block. We used the grid-search strategy for the optimization of hyperparameters. There are 64 convolution kernels with a size of 3 each. For the max-pool layer, the size of the max-pool windows is 2. The drop rate is set at 0.2 to avoid overfitting. The concatenate stage concatenates the three blocks of DCNNs to build a multiscale feature extractor. The BiLSTM stage applies a Bi-direction LSTM network to the input in order to collect long-term data dependency information between the data. The number of neurons is set at 64, and the drop rate is 0.2. After the BiLSTM stage, the data were flattened into one dimension by the flatten layer, followed by a fully connected layer. The fully connected layer consists of three full connections, which contain the number of neurons is 256,128,64, activated by ReLU function, and dropout with a probability of 0.5. Finally, the output layer calculates the probability score to indicate the likelihood of the site being modified with the Sigmoid function as follows:

22 $$\overset{\lower0.5em\hbox{$\smash{\scriptscriptstyle\frown}$}}{y} (x) = sigmoid(x) = \frac{1}{{1 + e^{ - x} }}$$

### Ensemble-based site prediction

**Fig. 8:**
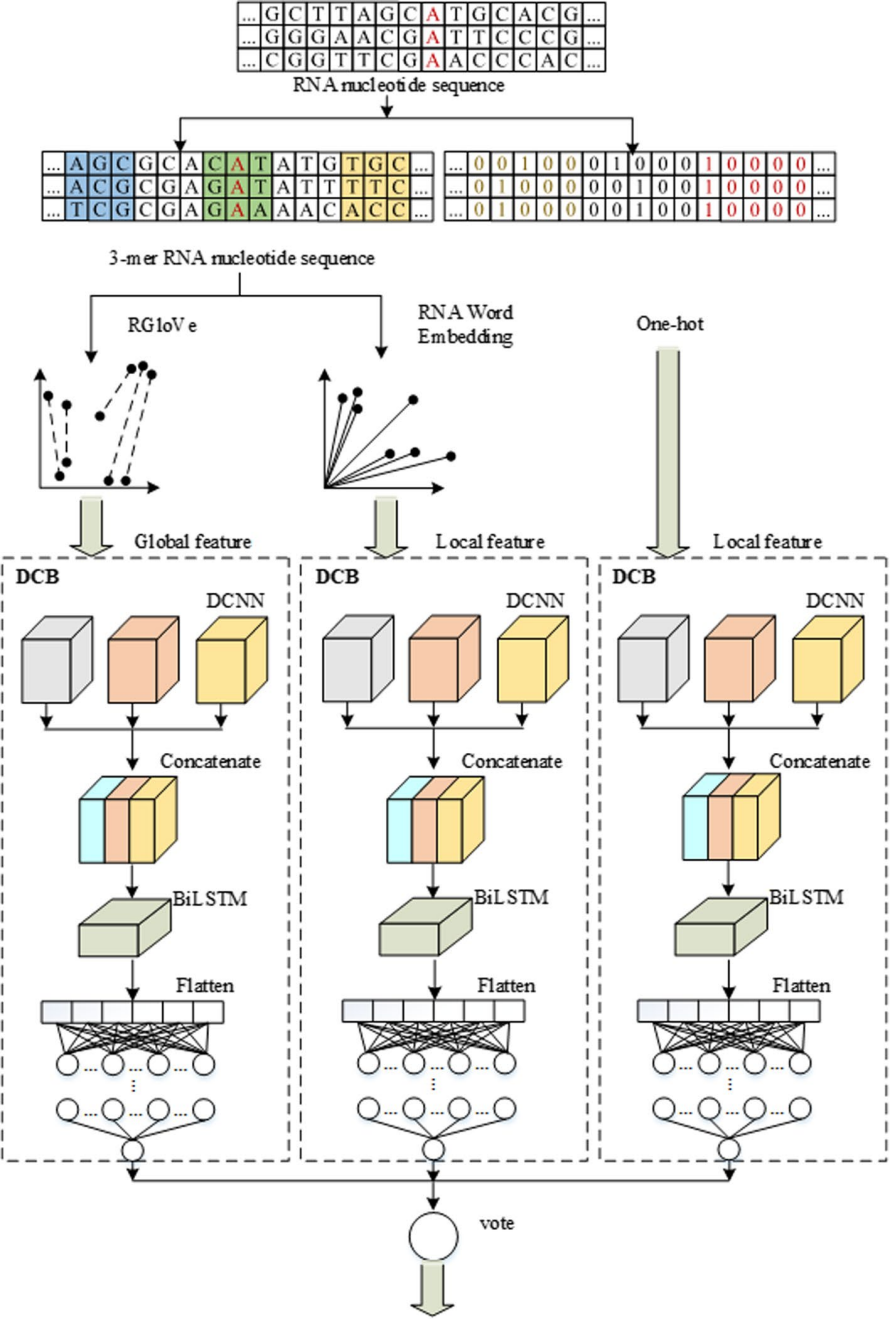
Structure of EMDLP predictor. The diagrams depicted our method's architecture. Three different DL classifiers predicted the methylation sequences and decided the final finding by a soft vote

Various encoding techniques will observe the sequences from various perspectives. RNA word embedding and One-hot encoding emphasize the local information, while RGlove employs global statistics to learn the global semantics. As a result, different predictors may have complementary impacts on prediction. Based on the DCB model, three predictors are constructed by RNA word embedding, One-hot encoding, and RGloVe. Finally, EMDLP was formulated with the three predictors above by a soft vote, as shown in Fig. 8.

## Supplementary Information

**Additional file 1.** Supplementary Figures.

## Data Availability

The data supporting the findings of the article is available at the webserver http://www.labiip.net/EMDLP/index.php (http://47.104.130.81/EMDLP/index.php). The code implemented to perform the analysis is deposited at https://github.com/whl-cumt/EMDLP.

## Abbreviations

RNA: Ribonucleic acid
m^6^A: N6-methyladenosine
m^1^A: N1-methyladenosine
CNN: Convolutional neural network
BiLSTM: Bidirectional long short-term memory
LSTM: Long short-term memory
RNN: Recurrent neural network
NLP: Natural language processing
DL: Deep learning
EMDLP: Ensemble multiscale deep learning model for RNA methylation site prediction
DCB: Dilated convolutional bidirectional long short-term memory network
DCNN: Dilated convolutional neural network
GloVe: Global vectors
Sn: Sensitivity
Sp: Specificity
ACC: Accuracy
Pre: Precision
MCC: Matthews correlation coefficient
AUROC: Area under the receiver operating characteristic
AUPRC: Area under the precision-recall curve
ENAC: Enhanced nucleic acid composition
