# Supplementary material for: EMDLP: Ensemble multiscale deep learning model for RNA methylation site prediction
Source: BMC Bioinformatics. 2022 Jun 8;23:221. doi: 10.1186/s12859-022-04756-1 (PMC9178860; doi:10.1186/s12859-022-04756-1)
Supplement: Supplementary file 1 — Additional file 1. Supplementary Figures. [file 12859_2022_4756_MOESM1_ESM.docx]

**EMDLP: Ensemble multiscale deep learning model for RNA methylation site prediction**

Honglei Wang^1,2,3^, Hui Liu1^1,2*^, Tao Huang^2^, Gangshen Li^1,2^, Lin Zhang^1,2^, Yanjing Sun^1,2*^

^1^ Engineering Research Center of Intelligent Control for Underground Space, Ministry of Education, China University of Mining and Technology, Xuzhou 221116, China

^2^ School of Information and Control Engineering, China University of Mining and Technology, Xuzhou 221116, China

^3^ School of Information Engineering, Xuzhou College of Industrial Technology, Xuzhou 221400, China

*To whom correspondence should be addressed.

**Additional File 1**

**Supplementary Figures**

**
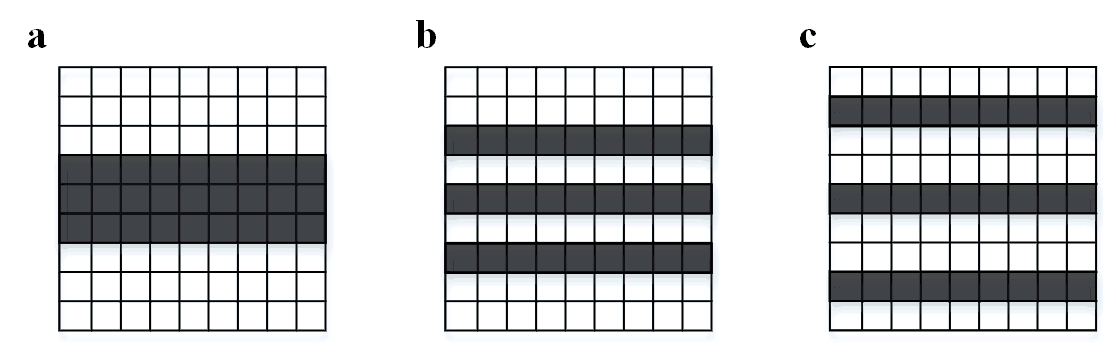
**

**Fig. S1** Different dilation rates in 1-D DCNN with a kernel size of 3. **a** DR=1. **b** DR=2. **c** DR=3.


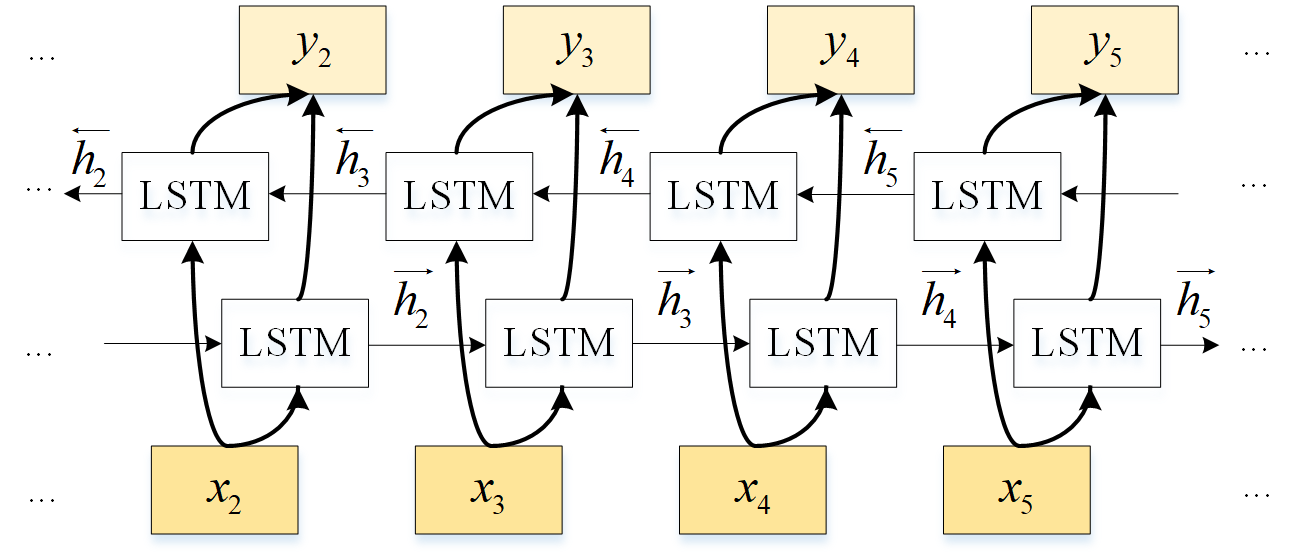


**Fig. S2** Diagram of BiLSTM model.


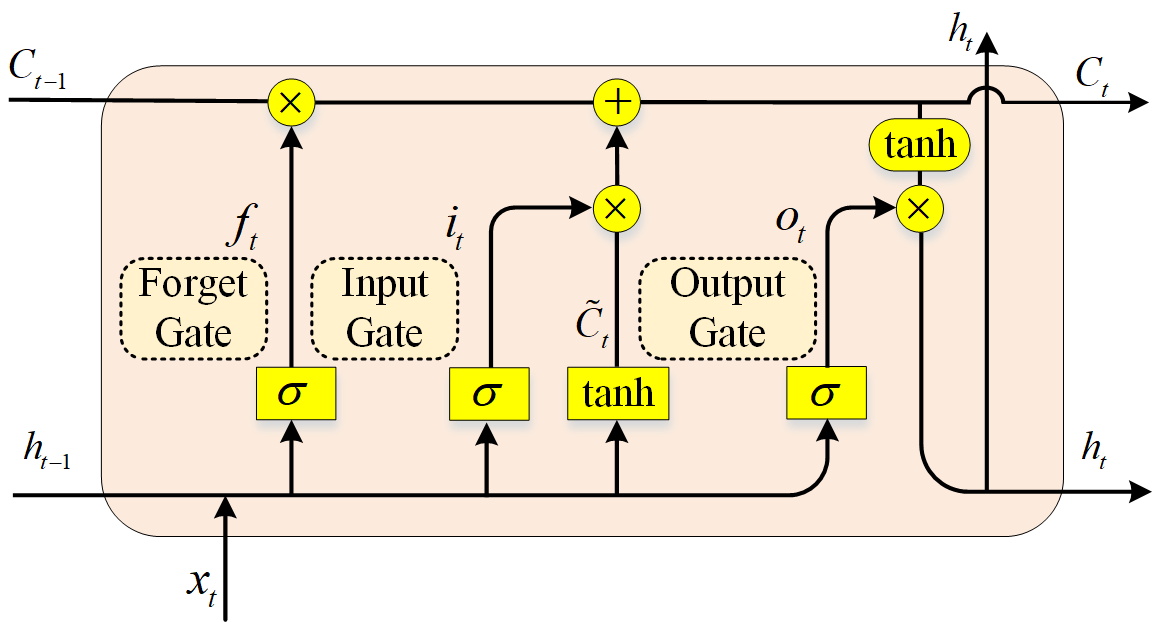


**Fig. S3** There are three gates in the LSTM internal department control model diagram.
